# DNA Methylation status of Wnt antagonist *SFRP5* can predict the response to the EGFR-tyrosine kinase inhibitor therapy in non-small cell lung cancer

**DOI:** 10.1186/1756-9966-31-80

**Published:** 2012-09-25

**Authors:** Jian Zhu, Yuyan Wang, Jianchun Duan, Hua Bai, Zhijie Wang, Lai Wei, Jun Zhao, Minglei Zhuo, Shuhang Wang, Lu Yang, Tongtong An, Meina Wu, Jie Wang

**Affiliations:** 1 Department of Thoracic Medical Oncology Peking University School of Oncology, Beijing Cancer Hospital & Institute, Beijing, 100036, China; 2 Laboratory of Immunology, National Eye Institute, National Institutes of Health, 31 Center Drive MSC 2510, Bethesda, MD, 20892-2510, USA

**Keywords:** DNA methylation, EGFR-TKI, Wnt antagonists, Non-small cell lung cancer

## Abstract

**Background:**

It is well known that genetic alternation of epidermal growth factor receptor (*EGFR*) plays critical roles in tumorgenesis of lung cancer and can predict outcome of non-small-cell lung cancer treatment, especially the EGFR tyrosine-kinase inhibitors (EGFR-TKIs) therapy. However, it is unclear whether epigenetic changes such as DNA methylation involve in the response to the EGFR-TKI therapy.

**Methods:**

Tumor samples from 155 patients with stages IIIB to IV NSCLC who received EGFR-TKI therapy were analyzed for DNA methylation status of Wnt antagonist genes, including *SFRP1*, *SFRP2*, *SFRP5*, *DKK3*, *WIF1*, and *APC*, using methylation specific PCR (MSP) method. EGFR mutations detections were performed in the same tissues samples using Denaturing High Performance Liquid Chromatography (DHPLC).

**Results:**

We found that Wnt antagonists tend to methylate simultaneously. Methylation of sFRP1 and sFRP5 are reversely correlated with EGFR mutation (P = 0.005, P = 0.011). However, no correlations of methylations of other Wnt antagonist genes with EGFR mutation were found. The patients with methylated *SFRP5* have a significant shorter progression free survival than those with unmethylated *SFRP5* in response to EGFR-TKI treatment (P = 0.002), which is independent of *EGFR* genotype.

**Conclusions:**

Patients with unmethylated *SFRP5* are more likely to benefit from EGFR-TKI therapy.

## Background

Lung cancer is the leading cause of cancer death worldwide [1]. NSCLC is the most common form of lung cancer, accounting for approximately 85% of lung cancer cases [2,3]. The efficacy of traditional chemotherapy has reached a plateau [4-6]. Therefore, new approaches are needed to improve the efficacy of lung cancer therapy. A number of targeted anticancer agents have been recently developed and approved for clinical use, among which the EGFR-TKI has been used as the first-line therapy for lung cancer patients with EGFR mutations [7-11].

*EGFR* gene product functions as a receptor tyrosine kinase that affects cell proliferation and survival by activating downstream signaling pathways. In 2004, three research groups reported that mutations in the tyrosine kinase domain of *EGFR* can predict the responses to TKIs in NSCLC patients [12-14], which enables the identification of patient populations that are more likely to benefit from TKI therapies and serves as the first step toward personalizing lung cancer therapy. However, according to the theory of “EGFR addition”, which refers to the dependency of cancer cells on *EGFR* mutation to maintain their malignant phenotypes [15], lung cancer patients harboring mutations in the tyrosine kinase domain of their *EGFR* genes should survive much longer, in response to the EGFR-TKI therapy, than the actual result. This suggested that *EGFR* mutation cannot explain all clinical outcomes of TKI therapy. At least 10 ~ 20% of patients with wild-type *EGFR* still significantly benefit from EGFR-TKI treatment, whereas around 10% of patients with mutated EGFR are resistant to the TKI therapy [10,16,17]. In addition, previous studies reported that both T790M mutation [18] and c-MET amplification [19] involved in acquired resistance of EGFR-TKI therapy. Therefore, factors in addition to *EGFR* genotype may also contribute to the response to EGFR-TKI therapy.

The Wingless-type (Wnt) signaling cascade is an important regulator of embryonic development [20]. Activation of Wnt signaling pathway leads to elevated expression of ß-catenin in cytoplasm, which in turn translocates to the nucleus, interacts with T cell factor/lymphocyte enhancer factor family, induces, downstream target genes that regulate cell proliferation and cancer progression. Aberrant activation of Wnt signaling pathway has been found in a number of tumors [21], which can be categorized into the following three common forms: 1) mutations in *APC* and/or *Axin*; 2) aberrant activation of Wnt signaling induced by activated *EGFR*[22]; 3) methylation of Wnt antagonists. Mutations of *APC* and/or *Axin* are rarely found in lung cancer patients. In addition, EGFR-TKI treatment blocks activation of EGFR in patients. Therefore, we hypothesized that the methylation of Wnt antagonists might significantly affect the responses to the EGFR-TKI therapy in NSCLC patients. Suzuki et al [23] analyzed the synchronous effects and correlations between Wnt antagonists and EGFR mutations and found that EGFR mutation was correlated with a good prognosis in tumors without methylated wnt antagonist genes.

In current study, we analyzed the methylation status of the CpG sites within Wnt antagonist genes, including *SFRP1*, *SFRP2*, *SFRP5*, *WIF1*, *DKK3*, *APC*, and *CDH1*, in 155 Chinese patients who received EGFR-TKI therapy and investigated potential clinical implication of the epigenetic regulation of Wnt antagonists.

## Methods

### Patients

155 patients were enrolled in current study. They were pathologically diagnosed as stage IIIB or IV NSCLC, with Eastern Cooperative Oncology Group performance (ECOG) status of 0 to 2; and received EGFR-TKI as either first- or second-line therapy at the Peking University Cancer Hospital between June 2006 and December 2009. The study was reviewed and approved by the Institutional Review Board at the Beijing Cancer Hospital. Written informed consent was obtained from all participants.

The smoking status of patients was decided during their first visit. A smoker was defined as the one who smoked more than 100 cigarettes in his/her life time. Patients were treated with either TKI therapy or platinum-based chemotherapy as the first line of treatment until their disease progressed, justified by imaging evidence or aggravated symptoms. The Response Evaluation Criteria in Solid Tumors (RECIST) [24] including progressive disease (PD), stable disease (SD), partial remission (PR) and complete remission (CR) was used to evaluate the drug response after patients received treatment every 6 weeks to 2 months. The objective response rate (ORR) was defined as the sum of PR and CR, while the disease control rate (DCR) was defined as the sum of SD, PR, and CR. Progression-free survival(PFS) was assessed from the beginning of therapy to disease progress or death from any cause. Overall survival(OS) was assessed from the beginning of first-line therapy until death from any cause.

### DNA extraction and methylation-specific PCR

Genomic DNA of tumor tissues from patients biopsied before TKI treatment were extracted using QIAmp FFPE DNA kit (Qiagen). The methylation status of the CpG sites within the gene loci of *SFRP1*, *SFRP2*, *SFRP5*, *WIF1*, *DKK3*, *APC*, and *CDH1* was decided by MSP assays as described previously [25-27]. Briefly, genomic DNA was treated with sodium bisulfite, followed by PCR amplifications using the primer pairs that can specific detect either the methylated or the unmethylated CpG sites. Genes were defined as methylated if the PCR products could be detected using the methylated DNA-specific primer pairs, while they were defined as unmethylated if the PCR products could only be detected using the unmethylated DNA-specific primer pairs. DNA from the human adenocarcinomic alveolar basal epithelial cell lines, A549 and A549/DDP, was used as the positive control for methylated DNA, while DNA from lymphocytes of healthy nonsmoking volunteers was used as the negative control. The methylation status results were confirmed by at least one repeat of the methylation-specific PCR assays. The following primers were used:

***sFRP1***:MethylatedF:5’-GTTTTCGGAGTTAGTGTCGCGC-3’,R:5’-ACGATCGAAAACGACGCGAACG-3’,UnmethylatedF:5’-GTAGTTTTTGGAGTTAGTGTTGTGT-3’,R:5’-ACCTACAATCAAAAACAACACAAACA-3’;***sFRP2***:MethylatedF:5’-TCGGAGTTTTTCGGAGTTGCGC-3’,R:5’-GCTCTCTTCGCTAAATACGACTCG-3’,UnmethylatedF:5’-GGTTGGAGTTTTTTGGAGTTGTGT-3’,R:5-CCCACTCTCTTCACTAAATACAACTCA-3’;***sFRP5***:MethylatedF:5’-TGGCGTTGGGCGGGACGTTC-3’,R:5’-AACCCGAACCTCGCCGTACG-3’,UnmethylatedF:5’-TGGTGTTGGGTGGGATGTTTG-3’,R:5’-CAACCCAAACCTCACCATACAC-3’;***DKK3***MethylatedF:5’-GGGGCGGGCGGCGGGGC-3’,R:5’-ACATCTCCGCTCTACGCCCG-3’,UnmethylatedF:5’-TTAGGGGTGGGTGGTGGGGT-3’,R:5’-CTACATCTCCACTCTACACCCA-3’;***WIF-1***MethylatedF:5’-CGTTTTATTGGGCGTATCGT-3’,R:5’-ACTAACGCGAACGAAATACGA-3’,UnmethylatedF:5’-GGGTGTTTTATTGGGTGTATTGT-3’,R:5’-AAAAACTAACACAAACAAAATACAAAC-3’;***APC***MethylatedF:5’-TATTGCGGAGTGCGGGTC-3’,R:5’-TCGACGAACTCCCGACGA-3’,UnmethylatedF:5’-GTGTTTTATTGTGGAGTGTGGGTT-3’,R:5’-CCAATCAACAAACTCCCAACAA-3’;***CDH-1***MethylatedF:5’-TGTAGTTACGTATTTATTTTTAGTGGCGTC-3’,R:5’-CGAATACGATCGAATCGAACCG-3’,UnmethylatedF:5’-TGGTTGTAGTTATGTATTTGTTTTTAGTGG-3’,R:5’-ACACCAAATACAATCAAATCAAACCAAA-3’.

### Mutation detection

The denaturing high-performance liquid chromatography (DHPLC) was used to detect mutations in the exon 19 and 21 of EGFR tyrosine kinase domains as described previously [28].

### Statistical analysis

All data were analyzed using SPSS (version 16.0). Chi-square and Fisher’s exact tests were used to assess the association between DNA methylation and EGFR genotypes. Multivariate analysis was performed using Cox proportional hazard regression model. The Kaplan-Meier method was used to determine the overall survival and progression-free survival curves. P value less than 0.05 was considered statistically significant.

## Results

### Characteristics of study patients

Table 1 summarized the demographic characteristics of 155 study patients, among which 118 cases were adenocarcinoma and 37 cases were non- adenocarcinoma (29 squamous carcinoma, 5 large cell carcinoma, and 3 adeno- squamous carcinoma cases). 60 of all patients received EGFR-TKI as the first-line therapy, while the rest had EGFR-TKI as the second- or more-line treatment. Among those 95 patients who had EGFR-TKI as the second- or more-line treatment, 63 patients took platinum-based chemotherapy as the first-line treatment. The median follow-up time for all patients was 22.4 months (from 2.4 to 77.2 months).

**Table 1:** Methylation and mutation profile of NSCLC

**Clinical characteristics (cases)**	**Methylation (%)**								**EGFR mutation (%)**
									
	**SFRP1**	**SFRP2**	**SFRP5**	**DKK3**	**WIF1**	**APC**	**CDH1**	**Any gene**	
*Gender*									
Male (74)	30 (40.5)	20 (27.0)	9 (12.2)	9 (12.2)	3 (4.1)	13 (17.6)	7 (9.5)	44 (59.5)	36 (48.6)
Female (81)	31 (38.3)	20 (24.7)	14 (17.3)	13 (16.0)	3 (3.7)	18 (22.2)	8 (9.9)	48 (59.3)	49 (60.5)
*Age*									
<65 (89)	33 (37.1)	21 (23.6)	10 (11.2)	12 (13.5)	3 (3.4)	16 (18.0)	7 (7.9)	48 (53.9)	56 (62.9)*
≥65 (66)	28 (42.4)	19 (28.8)	13 (19.7)	10 (15.2)	3 (4.5)	15 (22.7)	8 (12.1)	44 (66.7)	29 (43.9)
*Smoking*									
Never (93)	35 (37.6)	24 (25.8)	14 (15.1)	15 (16.1)	2 (2.2)	21 (22.6)	8 (8.6)	58 (62.4)	57 (61.3)*
Smokers (62)	26 (41.9)	16 (25.8)	9 (14.5)	7 (11.3)	4 (6.5)	10 (16.1)	7 (11.3)	34 (54.8)	28 (45.2)
*Histology*									
Adenocarcinoma (118)	46 (38.9)	30 (25.4)	16 (13.6)	16 (13.6)	4 (3.4)	21 (17.8)	14 (11.9)	72 (61.0)	65 (55.1)
Non-adenocarcinoma (37)	15 (40.5)	10 (27.0)	7 (18.9)	6 (16.2)	2 (5.4)	7 (18.9)	1 (2.7)	20 (54.1)	20 (54.1)
Total	61 (39.4)	40 (25.8)	23 (14.8)	22 (14.2)	6 (38.7)	31 (20%)	15 (9.7%)	92 (59.4%)	85 (54.8%)

*The frequency of this group is significantly higher than their counterparts.

### Epigenotype of Wnt antagonists in NSCLC

Genomic DNA was extracted from tumor tissues of all patients as described in the Method Section. The methylation status of Wnt antagonist genes including *SFRP1*, *SFRP2*, *SFRP5*, *WIF1*, *DKK3*, *APC*, and *CDH1*, defined as their epigenotype, was detected by Methylation Specific PCR Assays (examples were shown in Additional file 1: Figure S1A). The frequency of methylation events in Wnt antagonist genes in patients with different demographic characteristics was listed in Table 1. Interestingly, no significant difference in epigenotype of Wnt antagonist genes was found between male and female, among different age groups, between smokers and non-smokers, or between adenocarcinoma and non-adenocarcinoma cases.

Using DHPLC, we also detected EGFR activating mutations in exon 19 or 21 (the examples of wild type, mutated exon 19, and mutated exon 21 were shown in Additional file 1: Figure S1B, 1C, and 1D). Among the 155 patients, 85 (55.4%) carried mutations in either exon 19 or 21 of the EGFR genes (Table 1).Similar to the previous studies, we found that EGFR mutation rates were significantly increased among the patients younger than 65 years old (P = 0.02, Fisher’s exact test) and the patients who are nonsmokers (P = 0.04, Fisher’s exact test). EGFR mutation reversely correlates with sFPR1 methylation (P = 0.005) and sFRP5 (P = 0.011). We fail to find methylation of other wnt antagonist genes correlated with EGFR mutation (Table 2).

**Table 2:** P value among methylated genes and EGFR mutation

	**sFRP1**	**sFRP2**	**sFRP5**	**DKK3**	**WIF-1**	**APC**	**CDH-1**	**EGFR mutation**
sFRP1	NA	0.004	0.005	0.008	0.02	<0.0001	0.266	0.005
sFRP2	0.004	NA	<0.0001	<0.0001	0.007	<0.0001	<0.0001	0.854
sFRP5	0.005	<0.0001	NA	<0.0001	<0.0001	0.06	<0.0001	0.011
DKK3	0.008	<0.0001	<0.0001	NA	0.0001	0.006	<0.0001	0.489
WIF-1	0.02	0.007	<0.0001	<0.0001	NA	0.03	0.02	0.094
APC	<0.0001	<0.0001	0.06	0.006	0.03	NA	0.126	0.546
CDH-1	0.266	<0.0001	<0.0001	<0.0001	0.02	0.126	NA	0.592
EGFR	0.005	0.854	0.011	0.489	0.094	0.546	0.592	NA
mutation								

**Figure 1:**
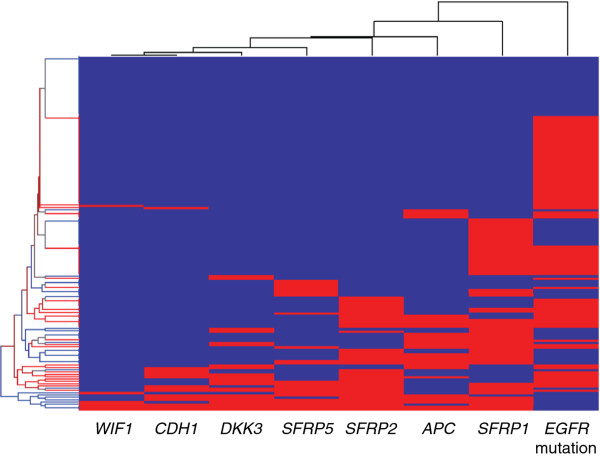
**Hierarchical clustering of Wnt antagonist DNA methylation status and EGFR genotype in 155 patients received EGFR-TKI therapy.** Red represents methylated gene or mutated EGFR, while blue represents unmethylated gene or wild-type EGFR, The figure of hierarchical clustering showed that the epigenotype of Wnt antagonist genes had similar patterns, which were different from the genotype of EGFR.

We next investigated whether the epigenotype of any Wnt antagonist genes correlated with the genotype of *EGFR*. Hierarchical clustering of the epigenotype of *SFRP1*, *SFRP2*, *SFRP5*, *WIF1*, *DKK3*, *APC*, and *CDH1*, as well as the genotype of *EGFR* (defined as “1” if mutation was detected in the exon 19 or 21, and as “0” if no mutation was detected) was generated using Partek Genomics Suite 6.5 (Partek Inc., MO). As shown in Figure 1, the epigenotype of Wnt antagonist genes had similar patterns, which were different from the genotype of *EGFR*. Therefore, our results suggested that the DNA methylation of Wnt antagonist might be independently regulated from the genotype of *EGFR*.

### Epigenotype of Wnt antagonist genes and clinical responses to TKI therapy

The RECIST was used to evaluate the clinical response of all patients to the TKI therapy. By the end of our study, 59 (38.1%), 53 (33.2%), 43 (27.7%) patients were defined with PD, SD, or PR, respectively. We then calculated the ORR and DCR and analyzed the difference between patient groups with different demographic characteristics, as well as with different genotypes of *EGFR* and epigenotypes of Wnt antagonist genes. As shown in Table 3, when only single factor was considered, the histology of the cancer (adenocarcinoma/nonadenocarcinoma), line treatment of TKI therapy (first line/not- first line), as well as smoking status (smoker/nonsmoker) significantly affected the ORR to the TKI therapy. Similarly, the gender (male/female), the histology of the cancer (adenocarcinoma/nonadenocarcinoma) as well as smo-king status (smoker/nonsmoker) were found to significantly affect the DCR of the TKI therapy. However, when all demographic characteristics were considered, only the histology of the cancer (P = 0.006, 95% CI, 1.712-26.057, multivariate logistic regression) was associated with ORR.

**Table 3:** Multivariate statistic of gender, age, histology, smoking status, treat line, EGFR mutation and SFRP5 methylation for objective response rate (ORR) and disease control rate (DCR)

**Variable**	**Objective response rate (ORR)**			**Disease control rate (DCR)**		
	**Univariate**	**Multivariate**		**Univariate**	**Multivariate**	
	**P value**	**P value**	**Hazard ratio (95% CI)**	**P value**	**P value**	**Hazard ratio (95% CI)**
**Gender (male / female)**	0.188	0.881	0.926 (0.337-2.542)	0.001	0.115	2.117 (0.834-5.734)
**Age (≤65 / >65)**	0.351	0.078	2.295 (0.912-5.772)	0.291	0.791	1.110 (0.515-2.393)
**Histology (adenocarcinoma / nonadenocarcinoma)**	0.002	0.006	6.680 (1.712-26.057)	0.049	0.244	1.663 (0.707-3.915)
**Line Treatment (first line / not-first line)**	0.016	0.078	2.184 (0.917-5.200)	0.940	0.491	0.756 (0.341-1.678)
**Smoking Status (smoker / nonsmoker)**	0.016	0.262	0.526 (0.171-1.617)	0.001	0.188	0.524 (0.200-1.371)
**EGFR Mutation (wide type / mutation)**	<0.0001	<0.0001	7.695 (2.895-20.454)	<0.0001	0.002	3.255 (1.540-6.881)
**SFRP5 Methylation (methylated / unmethylated)**	0.222	0.650	0.734 (0.193-2.788)	0.04	0.106	0.434 (0.158-1.193)

Previous studies have indicated that *EGFR* mutation significantly affected the ORR and DCR of the TKI therapy. Consistently, we found that the genotype of *EGFR* significantly affected the ORR (P < 0.0001, 95% CI, 2.895-20.454, multivariate logistic regression adjusted by gender, age, histology, line treatment, and smoking status) and the DCR (P = 0.002, 95% CI, 1.540-6.881, multivariate logistic regression adjusted by gender, age, histology, line treatment, and smoking status) (Table 3). Our results confirmed the higher response rate to the TKI therapy among patients with *EGFR* mutations as compared to the patients with wild-type *EGFR*.

Next, we investigated whether epigenotype of Wnt antagonists correlated with the clinical responses rate of the TKI therapy. Our univariate analysis identified the epigenotype of SFRP5 as the only potential factor significantly affecting DCR but not ORR (P = 0.04). However, the positive association of SFRP5 with DCR was not confirmed in multivariate analysis. When we sub-grouped patients based on their demographic characteristics, we found that SFRP1 methylation significantly reduced DCR in patients older than 65 (P = 0.038) and sFRP5 methylation significantly reduced DCR in patients suffered adenocarcinoma (P = 0.042).

### Epigenotype of Wnt antagonists and progression-free survival (PFS)

**Figure 2:**
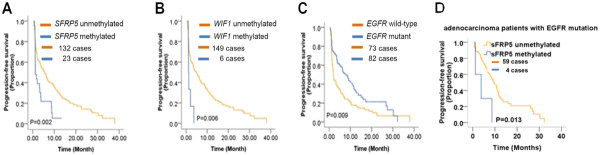
**Kaplan-Meier curves are shown comparing the progression free survival of patients with different epigenotypes of *SFRP5 *(A), *WIF1 *(B), different genotype of *EGFR *(C), or SFRP5 in adenocarcinoma with EGFR mutation group (D).**

We next analyzed whether the epigenotypes of Wnt antagonists could predict the PFS in response to the TKI therapy. The median PFS time in all patients was 5.1 months (ranging from 0.4 month to 38 months). Interestingly, as shown in Figure 2A, patients with methylated *SFRP5* gene had significantly shorter median PFS time (1.2 months, 95% CI, 0.5-1.9) as compared to those with unmethylated *SFRP5* gene (6.1 months, 95% CI, 4.4-7.8) (P = 0.002, Logrank Test). Similarly, patients with methylated *WIF1* gene had significantly shorter median PFS time (1.1 months, 95% CI, 95% CI, 1.0-1.2) as compared to those with unmethylated *WIF1* gene (5.4 months, 95% CI, 3.5-7.4) (P = 0.006, Logrank Test) (Figure 2B). We did not find association between epigenotype of other Wnt antagonists and PFS in response to the TKI therapy (Additional file 1: Figure S2 A-F). Moreover, after adjusted by age, gender, histology of the cancer, smoking status, and line of treatment, the methylation of *SFRP5* gene was still significantly associated with a shorter PFS (P = 0.008; harzard ratio, 2.165, 95% CI, 1.2-3.8; Cox proportional hazards models of survival analysis), while the methylation of WIF1 gene was no longer associated with a shorter PFS (P = 0.224; hazard ratio, 1.804, 95% CI, 0.7-4.7; Cox proportional hazards models of survival analysis) (Table 4). Taken together, our results suggested that the methylation status of *SFRP5* might be able to predict the PFS in response to the TKI therapy.

**Table 4:** Cox proportional hazard regression analysis of gender, age, histology, smoking status, EGFR mutation, WIF1 methylation and SFRP5 methylation for progression-free survival (PFS)

**Variable**	**P value**	**Hazard ratio (95% CI)**
*Smoking Status*	0.986	1.004
(smokers/nonsmokers)		(0.615-1.640)
*Histology*	0.689	0.915
(adenocarcinoma/Nonadenocarcinoma)		(0.592-1.414)
*Gender*	0.006	0.516
(male/female)		(0.322-0.826)
*Age*	0.456	0.858
(<65/>65)		(0.575-1.282)
*Lines of Treatment*	0.302	0.807
(first line/non-first line)		(0.537-1.213)
*EGFR Mutation*	0.024	0.656
(mutation/wide type)		(0.455-0.945)
*SFRP5 Methylation*	0.008	2.165
(methylated/unmethylated)		(1.226-3.823)
*WIF1 Methylation*	0.224	1.804
(methylated/unmethylated)		(0.697-4.674)

Similar to the previous discovery [27], we also found that the median PFS time for patients with *EGFR* mutations (8.3 months, 95% CI, 5.5-11.1) was significantly longer than the median PFS for patients with wide-type *EGFR* (2.0 months, 95% CI, 1.5-2.5) (P = 0.009, Logrank test) (Figure 2C). This is still valid when tested by Cox proportional hazards model of survival analysis (P = 0.024; hazard ratio, 0.656, 95% CI, 0.5-0.9; adjusted by age, gender, smoking status, histology of the cancer, and line of treatment). More interestingly, we found that in the subgroup of patients with adenocarcinoma and *EGFR* mutation, the ones with methylated *SFRP5* had a significantly shorter PFS (2.0 months), as compared to the ones with unmethylated *SFRP5* (9.0 months) (P = 0.013, Logrank Test) (Figure 2D).

### Epigenotype of Wnt antagonists and overall survival rate (OS)

**Figure 3:**
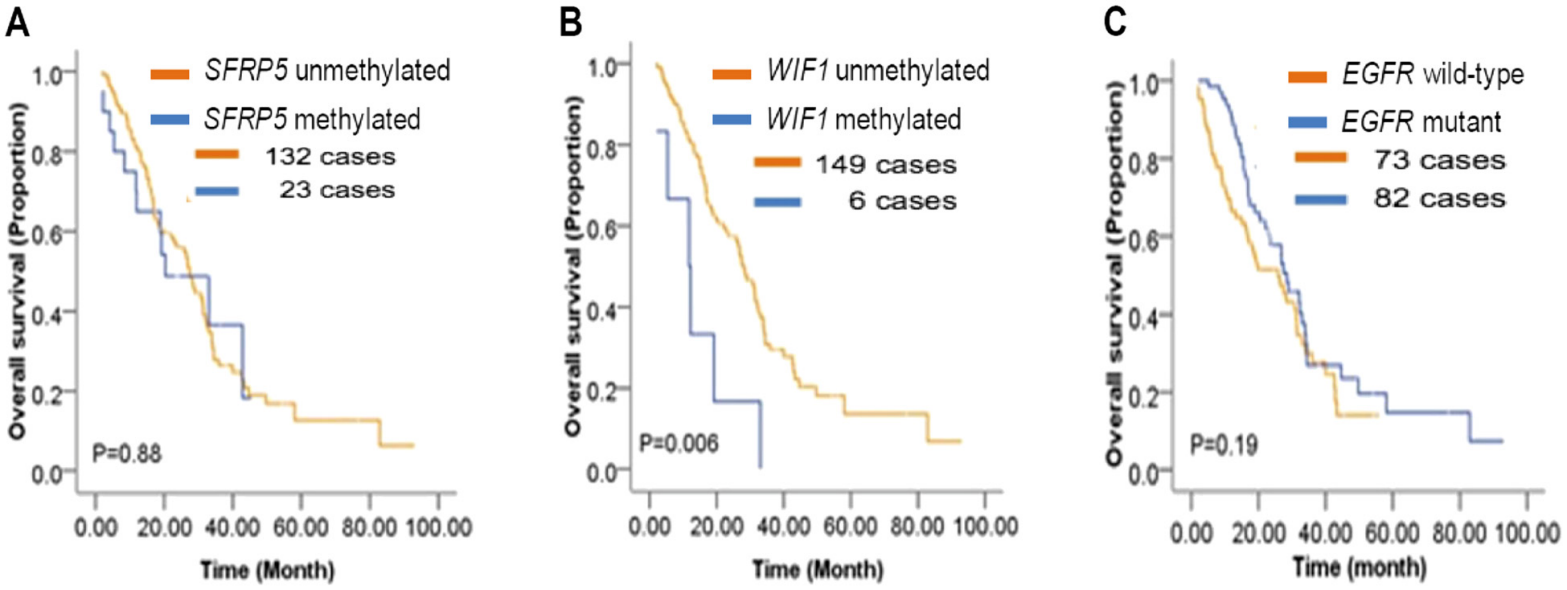
**Kaplan-Meier curves are shown comparing the overall survival of patients with different epigenotypes of *SFRP5 *(A), *WIF1 *(B), or different genotype of *EGFR *(C).**

To test whether the epigenotype of Wnt antagonists can predict the clinical outcome of the TKI therapy, we first investigated the association of DNA methylation of the Wnt antagonists and overall survival rate in our patient cohort. Nine patients (6.5%) were lost during the follow-up period of our study. The median OS time was 27.4 months (ranging from 3.0 to 93.1 months). Interestingly, patients with methylated *WIF1* genes had significantly reduced overall survival time (P = 0.006, Logrank Test) (Figure 3B), while the epigenotypes of *SFRP5* (Figure 3A), *SFRP1*, *SFRP2*, *DKK3*, *APC*, and *CDH1* (Additional file 1: Figure S3 A-E), as well as the genotype of *EGFR* (Figure 3C) were not associated with OS in our patients.

### Correlation between Wnt antagonist methylation and Progression-free survival in platinum-based chemotherapy

In order to decide if WIF-1 and sFRP5 are TKIs specific biomarkers related to PFS of TKIs treatment, we meanwhile analyzed the association of chemotherapy with the epigenotype of Wnt antagonists in 63 patients out of the whole group, who once took platinum-based chemotherapy as first-line treatment. We failed to find significant differences in PFS between patients with or without sFRP5 methylation (3.2 ms, 95% CI 2.01-4.5 vs 4.3 ms, 95% CI 2.5-6.2, respectively, P = 0.487). We did not find differences in PFS between patients with or without WIF-1 methylation (3.2 ms, 95% CI 1.89-4.67 vs 2.0 ms, 95% CI 1.71-2.36 P = 0.798) either. We accidentally found discrepancy in PFS between patients with or without sFRP1 methylation (1.8 ms,95% CI, 1.50-2.09 vs 3.0 ms 95% CI, 1.9-4.0, P = 0.017). However, this statistically significant difference in PFS remains limited for patients in clinical practice.

## Discussion

Recent studies have demonstrated that cancer is as much an epigenetic disease as it is a genetic disease (Iacobuzio-Donahue). Therefore, in addition to genetic alterations, changes in epigenetic features such as CpG DNA methylation status of specific gene loci also mark the progress of cancers. Our current study showed that methylation of Wnt antagonist *SFRP5* gene before treatment, independent of the genotype of EGFR gene, correlated with decreased progression free survival rate in NSCLC patients in response to the EGFR-TKI therapy. To our knowledge, this is the first report indicating that DNA methylation at specific gene loci in patient may predict drug response to the EGFT-TKI therapy.

Both genetic and epigenetic risk factors for NSCLC have been studied extensively. Suzuki et al [23] has reported that methylation of the Wnt antagonist *DKK3* correlated with low survival rate in NSCLC patients, despite of the different therapies patients received. However, in our study, we did not find significant difference in the EGFR-TKI responses between patient groups with or without methylated *DKK3* (Additional file 1: Figure S2 and S3). In contrast, our results suggested epigenotype of *SFRP5* provide better prognostic estimation for the EGFR-TKI response, comparing to other Wnt antagonists.

*SFRP5* is a member of the SFRP protein family containing a cysteine-rich domain homologous to the putative Wnt-binding site of Frizzled proteins. It acts as soluble antagonist of Wnt signaling and is highly expressed in the retinal pigment epithelium, and moderately expressed in the pancreas ("Entrez Gene: SFRP5 secreted frizzled-related protein 5"). Previous studies has identified association of *SFRP5* promoter hypermethylation with Acute myeloid leukemia [29], ovarian cancer [30], gastric cancer [31], oral squamous cell carcinoma [32], pancreatic cancer [33] and breast cancer [34].

We found that hypermethylation of *SFRP5* predicted worse outcomes of the EGFR-TKI therapy. Therefore, *SFRP5* DNA methylation status may serve as a prognostic molecular marker for appropriately predicting whether NSCLC patients would benefit from the EGFR-TKI therapy. Especially, it is interesting that in the subgroup with adenocarcinoma and EGFR mutation, patients with sFRP5 methylation have a significantly shorter PFS than those without sFRP5 methylation, While in nonsmokers without EGFR mutation, patients without sFRP1 methylation have a longer PFS compared with patients with its methylation(9.7 ms vs 2.0 ms, p = 0.05). Based on these results, we can make a hypothesis that activation of Wnt signaling by antagonist methylation could confer tumors the characters of stem cell, which consequently causes tumors resistant to EGFR TKIs therapy by generating acquired resistance, such as MET amplification or changes of PTEN tumor suppressor activity and so on. Further study is needed to validate this hypothesis.

## Conclusions

In conclusion, our study revealed that sFRP5 may be an independent factor affecting PFS during long time maintenance of TKIs therapy. Furthermore, the simple, PCR-based detection method of DNA methylation may be more feasible as clinical tests, compared to protein or RNA expression detection in clinics. Both general DNA methylation inhibitors and Wnt-pathway-targeting anticancer drugs are under development [35,36]. Our results that linked Wnt antagonist hypermethylation and EGFR-TKI response suggest that the treatment paradigm combining epigenetic drugs and EGFR-TKI may be a potential and attractive therapeutic option for patients with NSCLC.

## Abbreviations

EGFR: Epidermal growth factor receptor; EGFR-TKI: Epidermal growth factor receptor -tyrosine kinase inhibitors; MSP: Methylation specific PCR; Wnt: Wingless-type; ECOG: Eastern cooperative oncology group; ORR: Objective response rate; DCR: Disease control rate; PFS: Progression-free survival; OS: Overall survival; PD: Disease progression; CR: Complete response; PR: Partial response; SD: Stable disease; RECIST: Response evaluation criteria in solid tumors; HR: Hazard ratio.

## Competing interests

The authors declare that they have no competing interests.

## Authors’ contributions

JZ, YW carried out the molecular genetic studies; JD, MZ, ZW, JZ, SW, LY, TA, MW participated in Provision of study materials or patients and collection and assembly of data; LW, JZ, YW, HB and JW analyzed final data and JZ, YW, JW drafted the manuscript. All authors read and approved the final manuscript.

## Authors’ informations

Supported by grants from National Natural Sciences Foundation Distinguished Young Scholars (81025012), National Natural Sciences Foundation General Program (81172235), Beijing Health Systems Academic Leader (2011-2-22).

## Supplementary Material

Additional file 1 **Figure S1.** Methylated and unmethyalted bands of Wnt antagonist genes and wild/mutant EGFR. S1: The example graphs of methylated and unmethyalted bands of Wnt antagonist genes (A) and EGFR wild (B) and mutation types (C, D) by methylation specific PCR and DHPLC respectively. Figure S2 PFS with different epigenotypes of Wnt antagonist genes. Figure2S A-F.Kaplan-Meier curves of comparing the progression free survival of patients with different epigenotypes of SFRP1(A), SFRP2 (B), DKK3 (C), APC (D), CDH1 (E) and combination analysis (F). Figure S3 OS with different epigenotypes of Wnt antagonist genes. Figure3S A-F. Kaplan-Meier curves of comparing the overall survival of patients with different epigenotypes of SFRP1 (A), SFRP2 (B), DKK3 (C), APC (D), CDH1 (E) and combination analysis (F). Click here for file
