# Supplementary material for: DNA Methylation status of Wnt antagonist SFRP5 can predict the response to the EGFR-tyrosine kinase inhibitor therapy in non-small cell lung cancer
Source: J Exp Clin Cancer Res. 2012 Sep 25;31(1):80. doi: 10.1186/1756-9966-31-80 (PMC3524045; doi:10.1186/1756-9966-31-80)
Supplement: Additional file 1 — Figure S1. Methylated and unmethyalted bands of Wnt antagonist genes and wild/mutant EGFR. S1: The example graphs of methylated and unmethyalted bands of Wnt antagonist genes (A) and EGFR wild (B) and mutation types (C, D) by methylation specific PCR and DHPLC respectively. Figure S2 PFS with different epigenotypes of Wnt antagonist genes. Figure2S A-F.Kaplan-Meier curves of comparing the progression free survival of patients with different epigenotypes of SFRP1(A), SFRP2 (B), DKK3 (C), APC (D), CDH1 (E) and combination analysis (F). Figure S3 OS with different epigenotypes of Wnt antagonist genes. Figure3S A-F. Kaplan-Meier curves of comparing the overall survival of patients with different epigenotypes of SFRP1 (A), SFRP2 (B), DKK3 (C), APC (D), CDH1 (E) and combination analysis (F). [file 1756-9966-31-80-S1.ppt]

## Slide 1
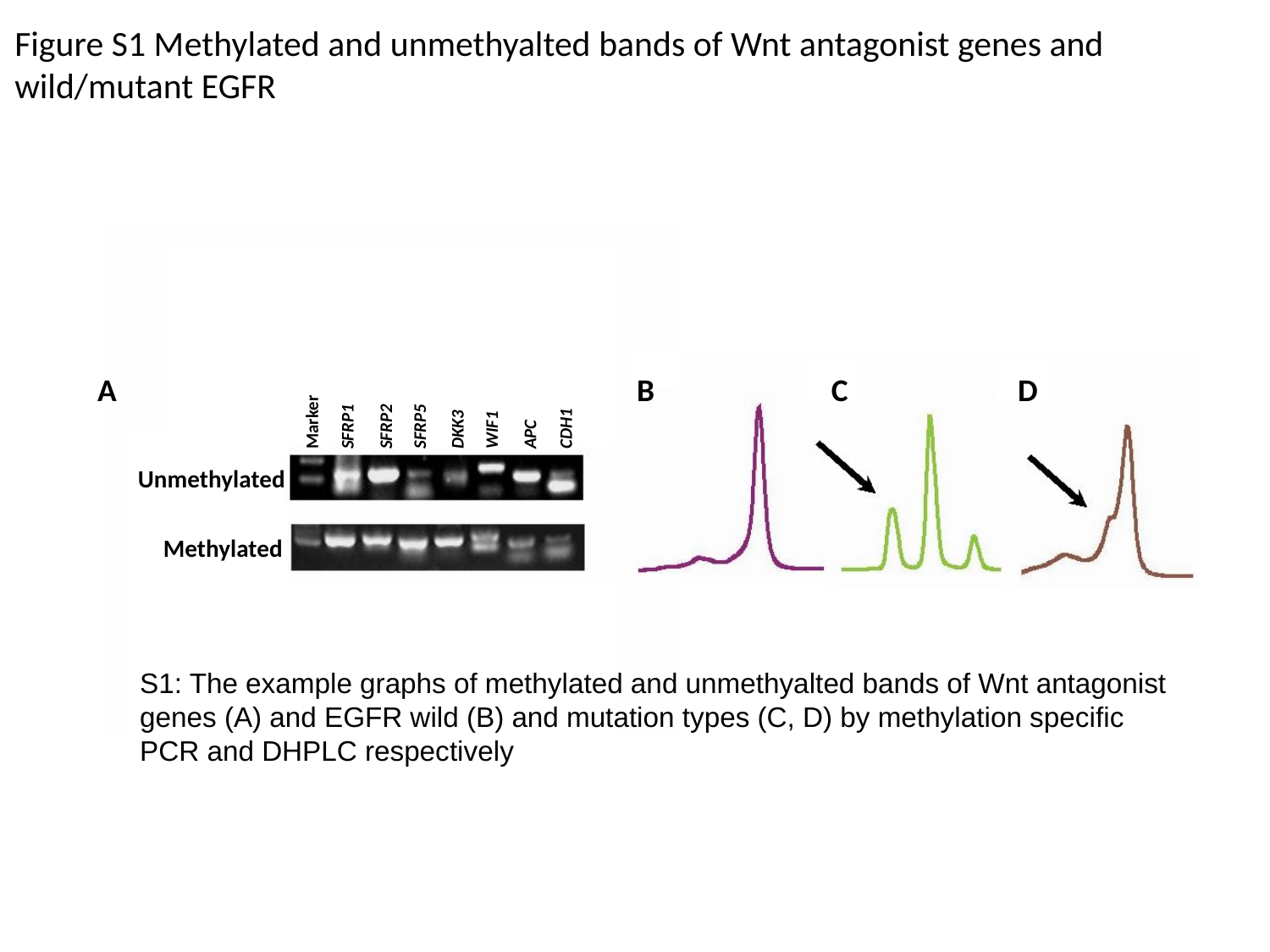

Figure S1 Methylated and unmethyalted bands of Wnt antagonist genes and wild/mutant EGFR
Marker
SFRP1
SFRP2
SFRP5
DKK3
WIF1
APC
CDH1
Unmethylated
Methylated
A
B
C
D
S1: The example graphs of methylated and unmethyalted bands of Wnt antagonist genes (A) and EGFR wild (B) and mutation types (C, D) by methylation specific PCR and DHPLC respectively

## Slide 2
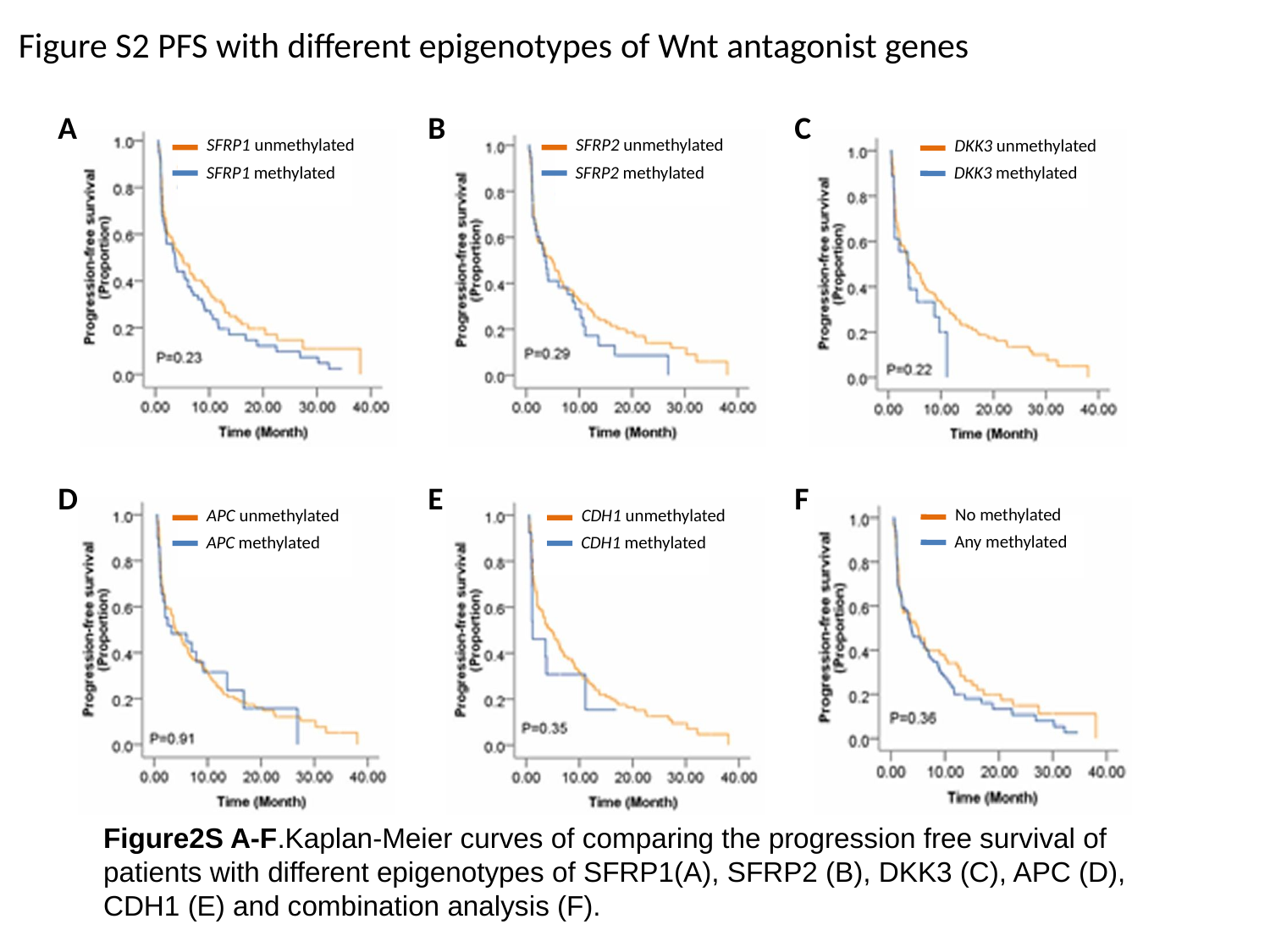

Figure S2 PFS with different epigenotypes of Wnt antagonist genes
A
B
C
SFRP1 unmethylated
SFRP1 methylated
SFRP2 unmethylated
SFRP2 methylated
DKK3 unmethylated
DKK3 methylated
D
E
F
No methylated
Any methylated
APC unmethylated
APC methylated
CDH1 unmethylated
CDH1 methylated
Figure2S A-F.Kaplan-Meier curves of comparing the progression free survival of patients with different epigenotypes of SFRP1(A), SFRP2 (B), DKK3 (C), APC (D), CDH1 (E) and combination analysis (F).

## Slide 3
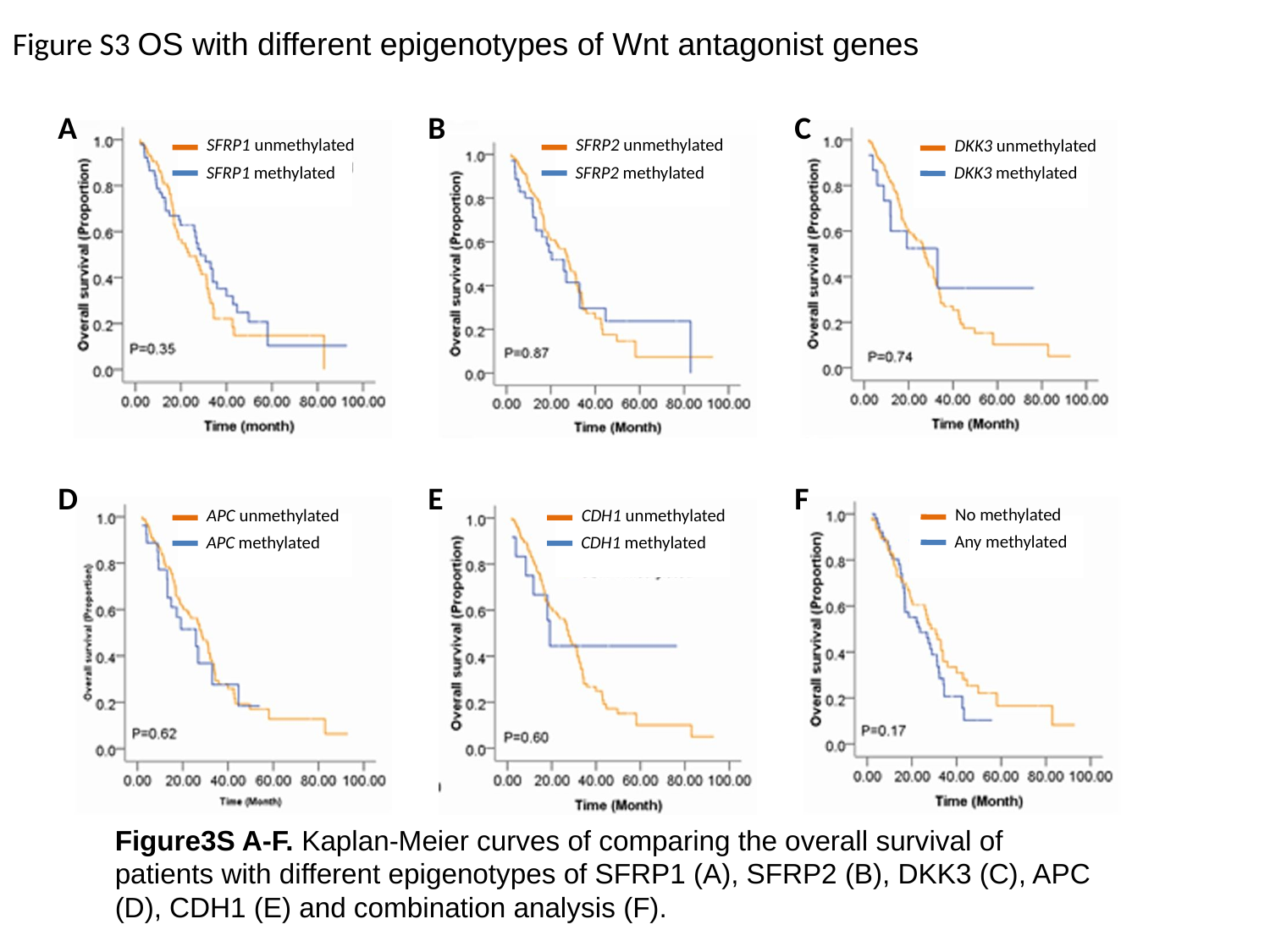

Figure S3 OS with different epigenotypes of Wnt antagonist genes
A
B
C
SFRP1 unmethylated
SFRP1 methylated
SFRP2 unmethylated
SFRP2 methylated
DKK3 unmethylated
DKK3 methylated
D
E
F
No methylated
Any methylated
APC unmethylated
APC methylated
CDH1 unmethylated
CDH1 methylated
Figure3S A-F. Kaplan-Meier curves of comparing the overall survival of patients with different epigenotypes of SFRP1 (A), SFRP2 (B), DKK3 (C), APC (D), CDH1 (E) and combination analysis (F).
